# Why are so many individuals with bulimia nervosa low in weight suppression?

**DOI:** 10.1186/s40337-025-01301-2

**Published:** 2025-06-05

**Authors:** Sarah M. Fisher, J. Ingrid Friedman, Michael R. Lowe

**Affiliations:** 1 Department of Psychological and Brain Sciences, Drexel University, 3201 Chestnut St., Room 119, Philadelphia, PA 19104 USA; 2 Osher Center for Integrative Medicine, University of California San Francisco, San Francisco, USA

**Keywords:** Bulimia nervosa, Developmental weight suppression, Weight suppression, Weight history

## Abstract

**Background:**

Weight suppression (WS) is associated with many problematic characteristics in individuals with bulimia nervosa (BN). It is theorized that WS contributes to eating disorder (ED) characteristics through the initiation of metabolic and appetitive responses that contribute to dysregulated food intake and weight gain. However, some individuals with BN exhibit little or no WS, and we investigated two possible explanations for this: that low-WS individuals were once weight-suppressed but regained most of the weight they previously lost, or that low-WS individuals never underwent the large weight losses that some of those with BN have shown.

**Methods:**

Participants were 453 female patients with BN. We used mixed-model ANOVAs to compare individuals with low and high WS on four weight variables (i.e., premorbid high, postmorbid high, postmorbid low, and current z-BMI). We conducted these analyses using a new, developmentally sensitive measure called developmental weight suppression (DWS).

**Results:**

Our results revealed strikingly different weight histories between low and high WS groups. The high WS groups displayed dramatic weight losses (and only partial weight regain), but the low WS groups demonstrated only modest weight losses and an overall pattern of weight gain over time.

**Conclusions:**

Individuals with BN and low WS do not show the same large and rapid z-BMI losses that are characteristic of most individuals with BN; rather, they show patterns of weight gain that are more characteristic of individuals with BED. Therefore, it may not be appropriate to include individuals who never lost significant weight in studies of WS in BN, as weight suppression would not be relevant to their presentation. Thus, there may be two groups of individuals with BN: those for whom weight suppression is a maintaining factor of binge eating, and those for whom it is not.

**Supplementary Information:**

The online version contains supplementary material available at 10.1186/s40337-025-01301-2.

## Abstract (plain language summary)

Weight suppression (WS), the difference between one’s highest past weight (or, for those with diagnosed eating disorders, their highest premorbid past weight) and current weight, has been found to be associated with symptom severity and treatment outcome among individuals with bulimia nervosa. It is theorized that these associations are due to metabolic and appetitive responses to being in a weight-suppressed state, yet some individuals with bulimia nervosa exhibit very low or no WS. This may be because they were once highly weight suppressed and have subsequently gained weight, or it could be that this subgroup was never significantly weight suppressed. We compared the weight histories of individuals with bulimia nervosa with low or no WS with individuals with bulimia nervosa with high WS. We found that the low WS subgroup has a dramatically different weight history than those high in WS, displaying only modest weight losses and a general pattern of weight gain over time. This suggests that the weight suppression model may not be applicable for this subgroup.

**Supplementary Information:**

The online version contains supplementary material available at 10.1186/s40337-025-01301-2.

## Background

Models of bulimia nervosa (BN) psychopathology can be broadly divided into those focusing on psychosocial variables and those focusing on biological or body mass variables [1–3]. In the latter domain, the investigation of weight history generally, and weight suppression in particular, has yielded an abundance of evidence that suggests that those with BN exhibit atypical weight patterns for their age and development. For example, many people with BN display elevated weights prior to the onset of their disorder [4]. Early research [5] found that women with BN were three times more likely than healthy controls to report childhood obesity prior to BN onset. Similarly, in a study by Shaw and colleagues, [6] female individuals with BN self-reported highest premorbid weights that were roughly 15.4 kg greater than the median weights for same-aged girls.

Despite commonly elevated premorbid weights in these individuals, most with BN present to treatment in a healthy weight range [7–9]. Given typically elevated weights prior to disorder onset and healthy weights when diagnosed, it follows that people with BN tend to undergo considerable weight loss during the development of their disorder. This difference between an individual’s highest past weight and their current weight has been operationalized as weight suppression (WS). Existing studies have found relatively high levels of WS in outpatients and inpatients with BN, as compared to their non-disordered peers [10–13].

Prior research has implicated WS in disorder onset, symptom severity, and treatment outcome in individuals with BN, in both cross-sectional and longitudinal studies. For example, higher WS was shown to predict BN onset at 3-year follow-up, [14] development of a bulimic syndrome after 10 years, [15] and greater bulimic symptoms 20 years later [16]. Additionally, higher WS has been associated with both greater fear of losing control over one’s weight [17] and greater baseline frequency of binge eating and purging in individuals with BN (even after controlling for BMI) [11, 18]. Furthermore, in a study of outpatients with BN, Butryn and colleagues [19] found that higher admission WS predicted lower rates of recovery and greater likelihood of treatment dropout. Finally, higher WS has been associated with greater weight gain in the short-term (i.e., during the course of treatment) [13, 20, 21] and long-term (i.e., over a 5-year period) [22] in patients with BN.

Although exact mechanisms underlying these associations remain unclear, hypotheses may be gleaned from the weight loss literature. Specifically, previous research suggests that large weight losses precipitate characteristic compensatory changes in metabolism and appetite (e.g., reduced energy expenditure, increased appetite), which are presumed to promote weight regain [23–27]. This paradox, wherein weight loss promotes weight regain, has previously been referred to as a “biobehavioral bind,” [28, 29] and similar processes may help explain the associations between WS and disordered eating in individuals with BN. That is, weight-loss induced increases in appetite might contribute to greater intake and instances of binge eating in individuals with BN, [30] leading to greater distress, restriction, and compensatory efforts in those individuals [28].

Thus, those with BN who are high in WS may have real reason to fear losing control over their weight (an association found in previous research [17]). If they have experienced greater drive to eat, increased instances of dysregulated eating, and/or weight gain in conjunction with their weight-suppressed state, their fear of losing control may be based in reality, not just in distorted cognitions [2]. Indeed, a large majority of individuals with BN report that dieting behaviors preceded binge eating [31, 32]. It may be the case that their initial dieting and subsequent weight loss contributed to the development of their binge eating. However, a minority of individuals with BN report the reverse order of behavior onset, with binge eating preceding dieting, suggesting that there may be two distinct groups of individuals with BN that differ significantly in the psychobiological mechanism underlying symptom onset [31, 33, 34]. Notably, this pattern of behavior onset is associated with binge eating disorder (BED), suggesting that there may be a subgroup of individuals with BN who more closely resemble those with BED, in that binge eating onset may not result from prior dieting-related weight loss but precede and contribute to substantial weight gain [35, 36].

At the same time, many studies on BN have found that some individuals report that they are currently at their highest past weight (i.e., show zero WS). Others report very low WS values [22, 37] that are likely insufficient to account for their regular binge eating or other eating disorder (ED) symptoms. Prior research in people with BN has also noted the presence of “negative WS” values, [38] which result from a self-reported highest past weight that is *lower* than one’s current weight. Because highest past weights are meant to be inclusive of current weights, negative WS is theoretically impossible, and these values have traditionally been corrected for or excluded from analyses entirely [38]. However, beyond simple errors in data reporting, negative WS may inadvertently capture individuals whose weight histories are characterized by weight gain, rather than significant weight losses. In other words, negative WS may be yet another indication of a subgroup of individuals with BN for whom WS does not seem to be a relevant maintaining factor.

The fact that some individuals with BN exhibit no or very low WS is an apparent paradox, since WS is posited to be a key driver for many characteristics and symptoms shown by those with BN [15, 18]. Individuals with BN who are not currently weight-suppressed, but who *are* bingeing and purging, raise the question of what might be driving their bingeing and purging if WS plays no role. Some researchers have suggested that drive for thinness may be a predisposing and maintaining factor for many individuals with BN, either alongside WS (if present) or as a solitary factor [39, 40]. Therefore, although WS may be a sufficient cause and/or maintaining factor in BN, it may not be a necessary one, and it is important to better understand instances in which it does not play such a role.

Examining the broader weight histories of individuals with low WS may provide important insights into this group. On one hand, it is possible that their low WS status is reflective of an overall weight history of relatively smaller weight losses, or even weight gains, over time. In other words, these individuals may have never experienced the same large and sustained weight losses that are thought to characterize most individuals with BN. In this case, low WS individuals might represent a distinct subgroup of people with BN, for whom weight suppression played little or no role in the etiology of the disorder and no role in its maintenance. On the other hand, it could be that individuals with low WS have exhibited significant WS at some point since reaching their adult height but then regained significant weight to a weight close to, or even beyond, their highest past weights. It is important to investigate these two scenarios, because they lead to quite different understandings of why a minority of those with BN consistently show low, or even “negative,” WS.

This study explored differences in weight history, including hallmark weight values (i.e., premorbid high weight, postmorbid high weight, postmorbid low weight, and current weight), between individuals with BN with low and high WS. There are two hypotheses as to why some individuals with BN have low (or no) WS. First, if individuals with low and high WS have experienced similar past weight losses but differ more in terms of their current weights, this may indicate that individuals who are currently low in WS have been highly weight suppressed in the past but have regained most of the weight they lost. This possibility would represent more of a “state” explanation, in that those currently low in WS were once in a state of WS but no longer are. Second, if individuals with low WS exhibit weight histories that show they have never been highly weight suppressed (and therefore, in terms of weight history, may resemble those with BED more than those with BN), then their weight histories may indicate more of a “trait” explanation, because such individuals would have never exhibited the high WS that characterizes the majority of those with BN.

Importantly, because flaws have been noted in the original formulation of the WS measure, [28, 41, 42] we duplicated all analyses using a new, more sensitive measure termed developmental weight suppression (DWS) [29, 43]. Whereas the traditional WS measure (TWS) uses raw weights in its calculation, DWS utilizes BMI z-scores (z-BMI) to account for the individual’s age and height at which hallmark weights were reached. Additionally, for individuals with eating disorders, DWS utilizes their *premorbid* past highest weight to avoid including any high weights that may have been the result of the eating disorder itself. For weight history comparisons, z-BMI scores were used for all hallmark weight variables in DWS analyses, while raw weights were used for TWS. Though this study examined both WS indices, as other recent studies have done, [29, 44] we focused on DWS in this paper, given recent evidence that DWS is a superior operationalization and measure of WS than TWS [9, 29, 44]. The results using TWS are reported in the Supplementary Materials.

## Methods

### Participants

Participants were females diagnosed with BN and admitted to a residential eating disorder treatment facility between April 2014 and May 2020. In cases of multiple admissions, only the first admission was used. To be included in this study, individuals must have consented to having their data used for research purposes, in addition to completing a historical weight questionnaire upon admission. Furthermore, given the variables needed for their respective calculations, to be eligible for TWS analyses, participants had to have their highest premorbid weight, highest postmorbid weight, and admission weight; to be eligible for DWS analyses, they had to have their highest premorbid weight, age, and height and admission weight, age, and height.

About 10% of new admissions to the treatment facility do not provide informed consent for research. Of 918 participants who did provide informed consent, 703 (76.6%) had all required TWS data, and 678 (73.9%) had all required DWS data. Following a tertile split (described further in Analytic Plan), the final samples comprised 467 participants for TWS and 453 participants for DWS; however, sample sizes may have been reduced for specific comparisons.

## Measures

### Weight history

Historical weights were assessed using the Dieting and Weight History Questionnaire (DWHQ), [45] a 15-item self-report measure. The DWHQ comprises 10 items pertaining to weight history. These items query age at which the first sign of an ED emerged (i.e., age of symptom onset), highest weight prior to that sign (i.e., premorbid high weight), highest weight after that sign (i.e., postmorbid high weight), lowest weight after that sign (i.e., postmorbid low weight), and estimated heights and ages at which each of these three weights occurred. Although questions may arise as to the validity of these recalled weights, prior research suggests they are credible [46, 47]. These studies were conducted in populations without EDs, but there is reason to believe that those with EDs may be even more accurate in recalling historical weights, given an overvaluation of weight and shape, tendencies for frequent self-weighing, [48] and the subsequently high emotional valence that is likely to be experienced around their weights [49]. Current weight and height were measured upon patients’ admission to the facility, and all weight and height data were reported in imperial units.

## z-BMI-Based weight history

All historical weight values from the DWHQ (i.e., premorbid high, postmorbid high, and postmorbid low) were converted into BMI, using their corresponding reported heights. Similarly, measured admission weight and height were used to calculate an admission BMI. Then, all four BMI values were transformed into z-BMI scores via a web-based user interface [43]. The resulting z-BMI scores represent an individual’s deviation from expected growth curves, according to CDC growth charts, at a given age.

Of note, CDC growth charts end at age 20. Therefore, for any current or historical weights occurring at age 21 or older, norms for age 20 were used to calculate respective z-BMI values. Support for this approach comes from the fact that CDC growth charts are intended to approximate optimal growth according to developmental needs, and little or no developmentally-driven changes are expected to occur after age 20 in women [50, 51].

## Developmental weight suppression

DWS was provided by the user interface (described above), which subtracted current z-BMI from *premorbid* high z-BMI and reset any negative values to 0, consistent with the proposed formulation [43].

### Procedure

Prior to admission to the clinic, an initial intake was conducted by a trained staff member over the phone to assess ED symptoms and preliminary diagnoses, including co-occurring psychiatric disorders, in accordance with criteria set forth by the Diagnostic and Statistical Manual of Mental Disorders (DSM). Patients admitted to the clinic prior to mid-December 2014 were assessed using the fourth edition of the DSM (DSM-IV), [52] and those admitted after that time were assessed using the fifth edition (DSM-5) [53]. Diagnoses were then confirmed upon admission by a psychiatrist using a semi-structured clinical interview based on DSM diagnostic criteria. Weight and height were measured using an electronic medical scale and stadiometer, and self-report measures (including the DWHQ) were administered following collection of informed consent. All research activities were approved (deemed exempt, IRB protocol #19172) by Drexel University’s institutional review board, in addition to the research department housed at the treatment center.

## Analytic plan

After calculating DWS, a tertile split was used to derive low and high groups; only the participants in the lower and upper tertiles were included in analyses. This method was deemed appropriate based on prior findings by Lowe and colleagues, [37] which suggested that WS levels in their bottom tertile of patients with BN were indeed low and comparable to those found in individuals without EDs. Hereafter, the “DWS sample” will refer to those individuals captured within the low or high tertile groups according to DWS. The use of a tertile split ensured the formation of two WS groups who were (as shown in the Results section) in fact quite low or high in WS.

Because participants were already recruited prior to analyses, sensitivity (rather than power) analyses were conducted using G*Power to determine the minimum effect size required to achieve statistical significance within our established samples. Given a desired power of 80%, we confirmed sufficient sensitivity to detect small effects (*f* < 0.10) at the 0.05 alpha level.

Mixed-model ANOVAs were used to assess differences in weight history between low and high WS groups. One DWS variable (postmorbid low z-BMI) was winsorized to the 95th percentile to address extreme outliers. Acceptable normality, skewness, and kurtosis were then confirmed for each variable. The assumption of sphericity was tested through Mauchly’s W and found to be violated (*p* <.001); therefore, our final results are reported with the Greenhouse-Geisser correction. Finally, Bonferroni post-hoc tests were conducted to probe for the source(s) of significant interactions.

## Results

Table 1 shows participant characteristics for the DWS sample (see SM.3 for the TWS sample). Most participants (78.6%) were White. On average, participants presented to the clinic as adults and reported experiencing their first ED sign as adolescents. Comparing low and high DWS tertile groups on demographic variables (i.e., age at admission, treatment length of stay, and age of symptom onset) resulted in no significant differences (*p*s > 0.05).

**Table 1:** Participant characteristics for the developmental weight suppression sample

	DWS			
	*n*	Min	Max	*M* (*SD*)
Age of symptom onset	453	2	38	14.6 (4.2)
Age at admission	453	14	65	25.8 (10.2)
Treatment length of stay, in days	452	4	99	28.8 (11.6)
*Ethnicity*	*n* (%)			
White	356 (78.6)			
African American	11 (2.4)			
Asian or Pacific Islander	12 (2.6)			
Hispanic	30 (6.6)			
Native American	5 (1.1)			
Other	17 (3.8)			
Multiracial	17 (3.8)			
Total	448 (98.9)			

Note. Age of symptom onset was self-reported age at which first eating disorder sign was experienced, derived from the Dieting and Weight History Questionnaire (DWHQ). DWS = developmental weight suppression

### Weight history analyses

Because past research supports the finding that DWS is a superior measure of weight suppression compared to TWS, we detail the weight history analyses using DWS below and report the analyses using TWS in the Supplementary Materials (SM.4).

**Fig. 1:**
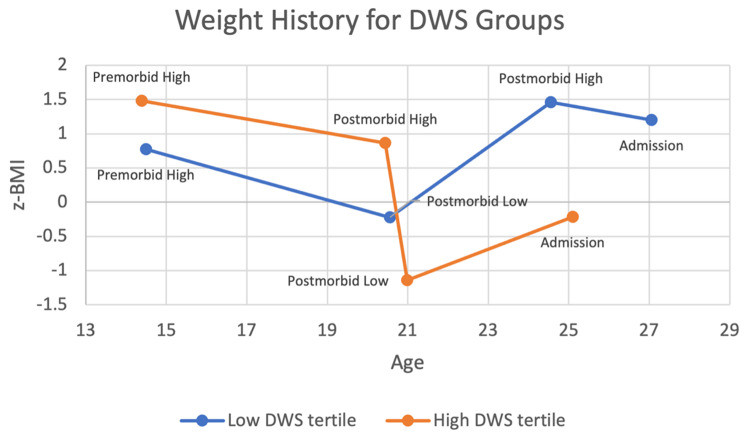
z-BMI-based weight history by age for low and high DWS groups. Note: Of those included in weight history analyses among the DWS sample (*n* = 368), age of symptom onset was 14.6 (*SD* = 4.0) for low DWS and 14.7 (*SD* = 4.5) for high DWS

A 2 × 4 mixed-model ANOVA was used to examine differences in weight history between low and high DWS groups. There was a significant interaction between z-BMI-based weight history category and tertile group, *F*(1.96,717.75) = 191.31, *p* <.001, *η*_*p*_^*2*^ = 0.34 (see Fig. 1) as well as significant main effects of z-BMI-based weight history category, *F*(1.96,717.75) = 691.48, *p* <.001, *η*_*p*_^*2*^ = 0.65, and tertile group, *F*(1,366) = 42.30, *p* <.001, *η*_*p*_^*2*^ = 0.10.

Bonferroni post-hoc tests revealed a number of significant within-group comparisons. First, for both low and high DWS groups, postmorbid low z-BMIs were significantly lower than all other hallmark weights (i.e., premorbid high, postmorbid high, and admission z-BMI; *p*s < 0.001). Second, for both groups, admission z-BMI was significantly lower than postmorbid high z-BMI (*p*s < 0.001), but the difference was descriptively much greater for the high DWS group than the low DWS group (mean difference of -1.10 z-BMI units vs. -0.26 z-BMI units, respectively). Third, whereas the high DWS group presented at z-BMIs that were significantly lower than their premorbid high z-BMIs (mean difference of -1.69 z-BMI units; *p* <.001), the low DWS group presented at z-BMIs that were significantly *higher* than their premorbid high z-BMIs (mean difference of 0.43 z-BMI units; *p* <.001). Thus, the high DWS group was substantially weight-suppressed compared to both their premorbid and postmorbid high z-BMIs, while the low DWS group was minimally weight-suppressed compared to their postmorbid high z-BMIs and negatively weight-suppressed compared to their premorbid high z-BMIs (i.e., their current z-BMI was higher than their highest past z-BMI). Furthermore, whereas within the high DWS group, postmorbid high z-BMI was significantly lower than premorbid high z-BMI (mean difference of -0.62 z-BMI units; *p* <.001), the low DWS group surpassed their premorbid high z-BMIs postmorbidly.

Between-group comparisons indicated that the high DWS group had higher premorbid high z-BMIs, and lower admission z-BMIs, than the low DWS group (*p*s < 0.001; see Table 2). Thus, the high WS levels in the high DWS group seemed to stem from *both* higher premorbid high z-BMIs and lower admission z-BMIs. Furthermore, the high DWS group was found to have significantly *lower* postmorbid low and postmorbid high z-BMIs than the low DWS group. Thus, the DWS formulation resulted in a high DWS group whose postmorbid z-BMIs (i.e., postmorbid high, postmorbid low, and admission) were all significantly lower than those found within the low DWS group, while their premorbid high z-BMIs were significantly higher.

**Table 2:** Weight history comparisons (in z-BMI units) between low and high developmental weight suppression groups

z-BMI categories	High DWS, M (SD)	Low DWS, M (SD)	Mean Difference (Low - High)	*p*
Premorbid high z-BMI	1.5 (0.8)	0.8 (0.9)	-0.7 (0.9)	< 0.001
Postmorbid high z-BMI	0.9 (0.9)	1.5 (0.8)	0.6 (0.1)	< 0.001
Postmorbid low z-BMI	-1.1 (1.2)	-0.2 (1.4)	0.9 (0.1)	< 0.001
Admission z-BMI	-0.2 (0.9)	1.2 (0.8)	1.4 (0.1)	< 0.001

Note: DWS = Developmental Weight Suppression; z-BMI = BMI z-score

### Post-hoc age analyses

When analyzing weight history data, apparent differences emerged between the two groups in age at which each of the hallmark weights was reached (see Fig. 1). Because the timing and order of these weights might contribute important information to an understanding of overall weight trajectories between the two groups, we decided to engage in post-hoc analyses examining age at which each hallmark weight occurred. For these analyses, we applied the same ANOVA model as used for weight history to assess differences in age between the two groups. When comparing DWS groups on age at which each z-BMI-based weight occurred, we found a significant interaction between age category (i.e., premorbid high, postmorbid high, postmorbid low, and admission) and tertile group, *F*(2.12,791.42) = 13.49, *p* <.001, *η*_*p*_^*2*^ = 0.35, as well as significant main effects of age category, *F*(2.12,791.42) = 301.56, *p* <.001, *η*_*p*_^*2*^ = 0.45, and tertile group, *F*(1,373) = 4.99, *p* =.03, *η*_*p*_^*2*^ = 0.01.

Bonferroni post-hoc analyses revealed a clear pattern within the low DWS group, wherein postmorbid low z-BMIs were experienced an average of 4.0 years *earlier* than postmorbid high z-BMIs (*p* <.001); however, within the high DWS group, postmorbid low z-BMI was experienced 0.6 years *later* than postmorbid high z-BMI, and these ages were not significantly different from each other (*p* = 1.00). Furthermore, whereas the majority of participants in the low DWS group (73.5%) reported experiencing their postmorbid low z-BMI prior to their postmorbid high z-BMI, the majority of participants in the high DWS group (59.3%) reported experiencing their postmorbid *high* z-BMI first. Thus, the order in which postmorbid low and postmorbid high z-BMIs were experienced for the majority of participants was reversed for the two groups.

## Discussion

This study explored why some individuals with bulimia nervosa (BN) present to treatment with low weight suppression (WS). On one hand, these individuals may have been highly weight suppressed at one point but then regained most, or all, of the weight that they had lost. On the other hand, they may never have been highly weight suppressed, and their weight histories may differ from the majority of individuals with BN, who usually show a major weight loss early in the development of their disorder [6]. This study found stronger support for the latter hypothesis, both in analyses using the traditional measure of WS and in those using the newer, empirically superior developmental measure of WS [23] (for details on the minor differences in these two patterns of results, see SM.5 in the Supplementary Materials). Low WS individuals never experienced the dramatic weight loss and subsequent WS that the high WS individuals did. This finding supports the idea that a large and rapid weight loss plays a major role in the etiology of many, but not all, individuals with BN. For those with low WS, this lack of a large weight loss, combined with the finding that those with low WS have higher z-BMIs at admission than they do premorbidly, suggests that WS may not meaningfully contribute to the onset and maintenance of binge eating and purging in this group.

Both the low WS and high WS groups had above-average premorbid z-BMIs at around age 14, which is in line with past research supporting the idea that above-average premorbid weights are a reliable risk factor for the later development of BN [4, 5, 54]. Additionally, both groups showed similar, statistically and clinically significant, declines in z-BMI between the approximate ages of 14 and 21, which is a novel finding. Although the absence of intermediate z-BMI values makes interpreting this finding difficult, it is possible that both groups show a prodromal heightened concern about their elevated z-BMIs and act on this concern to reduce their z-BMIs over time, either before or during the process of developing their eating disorder. Such a decline in z-BMI would not be expected in the general population, because adolescents and adults with elevated BMIs/z-BMIs at a given point in time tend to gain more weight, rather than lose weight, relative to those with lower BMIs/z-BMIs [55, 56].

After this timepoint, however, the two groups’ weight trajectories diverged dramatically. The high WS group lost weight at a prodigious rate, losing a mean of 20.9 kg over an average of 7.2 months (between their postmorbid high z-BMI at age 20.4 to their post-morbid low z-BMI at age 21.0). In z-BMI terms, these individuals went from somewhat overweight (z-BMI average of 0.9) to substantially underweight (z-BMI average of -1.1). In contrast, the low WS group experienced substantial weight gain to a postmorbid high z-BMI that was significantly higher than their premorbid high z-BMI. Between their postmorbid low z-BMI at age 20.6 and their postmorbid high z-BMI at age 24.6, they gained an average of 1.7 z-BMI units. If one drew a best-fitting regression line through the four weight history data points for the low and high WS groups, it would show a clear cross-over interaction, reflecting the very different directions of weight change in the two groups. Further, this sharp interaction exists despite essentially parallel z-BMI trajectories for the first seven years.

If the high WS group represents the most common body mass pattern shown by those with BN, [10, 11, 12, 13] the low WS group shows an opposite pattern that is more similar to those with binge eating disorder (BED), who do not typically show a large weight loss prior to developing binge eating and more often report that binge eating preceded dieting [35, 57, 58]. Previous literature has suggested the existence of two subgroups within the BN and BED populations, one in which individuals restrict their eating (and lose a significant amount of weight) prior to the onset of binge eating, and one in which the onset of binge eating occurred before the onset of restriction [3, 35]. Thus, the existence of a “traditional BN subgroup” and a “traditional BED subgroup” is not unique to the current sample. This pattern is consistent with a finding by Stice and colleagues, [14] who showed that WS increased the odds of developing BN, but not BED. However, despite these proposed similarities, a notable difference between those with BN and low WS in our sample and those with BED is the presence of sufficiently frequent compensatory behaviors to meet the diagnostic threshold for BN.

When interpreting these weight trajectories, it is important to note that the distinction between a “premorbid” and a “postmorbid” weight was based upon a participant’s own recollection of when the first sign of their eating disorder emerged, as well as what they considered to be the “first sign.” In other words, because our premorbid-postmorbid distinction relied on self-reported age at which each participant experienced their first sign of their impending eating disorder, it is possible that this distinction was not valid. This self-reported age of symptom onset (around 14) is likely earlier than the age at which their symptoms culminated in a DSM-defined eating disorder. Because historical symptom frequency data were not available, the results that we report on premorbid and postmorbid weight trajectories can only be interpreted relative to the fact that participants, not professionals, were the ones that defined the onset of their eating disorder. However, given that all participants responded to the same self-report item means that the two groups can still be meaningfully compared on the ages at which they report the hallmark weights examined.

Although this was not a goal of the present study, an interesting and unexpected finding was the relatively early age at which the first sign of an ED was reported (i.e., 14.6 years). Past research has found that BN tends to onset in the mid-to-late teenage years (e.g., 19.5 years in one study by Favaro and colleagues; [59] 17.4 years in a study by Nakai and colleagues [60]), several years after the first ED sign was reported by our participants. At least three influences might have produced this finding. First, the timing of this first sign of their ED could reflect the impact of puberty, which past research has suggested may activate genetic influences on disordered eating [61]. Second, it might reflect the fact that both low and high WS groups’ z-BMIs were above average at this time. Third, a substantial increase in fat mass occurs during puberty. Thus, it may be that individuals who eventually develop BN start to react to their heightened, post-puberty z-BMIs in a way that gradually reduces their z-BMIs over time, well before their symptoms evolve into a full-fledged BN diagnosis.

The fact that only self-report data were available for historical weight/z-BMI values and respective ages represents a limitation to the current study. Although prior studies have found strong associations between actual and recalled weights in both non-disordered [46, 47 and disordered^57^] populations, we cannot be certain as to the accuracy of recall for the current sample. Future research should, where possible, utilize objective weight data (e.g., by way of growth charts) to address the potential limitations inherent in recall and self-report. Additionally, as our understanding of participants’ weight trajectories was limited to four z-BMI data points, the use of growth charts may provide more data points. This would be especially beneficial if an individual has experienced numerous fluctuations between their highest and lowest weights, which could be clinically significant. Participants reported historical weight/z-BMI values which occurred at different ages, representing a potential weakness in that there may be differences partially attributable to the age at which a participant reached a certain milestone weight. Having additional data points from growth charts may also facilitate the inclusion of standardized data points that hold age constant across all participants. Of note, CDC growth charts end at age 20. We therefore used norms for age 20 to calculate z-BMIs for ages 21 and older, although it may be that there are expected changes in BMI throughout the lifespan that would have been better accounted for if growth charts spanning beyond age 20 were available. Other limitations are that our sample included a predominantly White, female-only sample, which may limit generalizability, and that a formal semi-structured interview to determine diagnoses (i.e., the EDE) was not utilized by the treatment clinic.

## Conclusions

This study provides the first investigation into the weight history of individuals with BN and low WS. Individuals with BN and low WS demonstrated markedly different weight histories compared to their high WS counterparts. Those with low WS did not show the same large and rapid z-BMI losses that are characteristic of most individuals with BN; rather, they showed overall patterns of weight gain over development that may be more similar to the weight histories seen in individuals with BED [35, 56]. Thus, it may not be appropriate to include individuals with very low WS in studies examining WS in patients with BN, if the reason for their low WS is that they never experienced a large, rapid weight loss during the development of their eating disorder. Future research should examine whether low-WS individuals with BN do indeed more closely resemble those with BED (e.g., in weight history, ED psychopathology, or psychobiological mechanisms underlying their symptoms), despite their BN diagnoses. There may be other factors causing and/or maintaining binge eating and purging in this group, and it is important that future research examine what these other factors may be.

## Electronic supplementary material

Below is the link to the electronic supplementary material.

Supplementary Material 1

## Data Availability

The datasets generated and/or analyzed during the current study are not publicly available due to privacy or ethical restrictions but are available from the corresponding author on reasonable request.

## Abbreviations

BED: Binge eating disorder
BMI: Body mass index
BN: Bulimia nervosa
CDC: Center for Disease Control
DSM: Diagnostic and Statistical Manual
DWHQ: Dieting and Weight History Questionnaire
DWS: Developmental weight suppression
ED: Eating disorder
TWS: Traditional weight suppression
WS: Weight suppression
z-BMI: Body mass indez z-score
