# Supplementary material for: Why are so many individuals with bulimia nervosa low in weight suppression?
Source: J Eat Disord. 2025 Jun 5;13:105. doi: 10.1186/s40337-025-01301-2 (PMC12142883; doi:10.1186/s40337-025-01301-2)
Supplement: Supplementary file 1 — Supplementary Material 1 [file 40337_2025_1301_MOESM1_ESM.docx]

**Supplementary Materials**

**SM.1 TWS Calculation**

TWS was calculated by subtracting an individual’s current (i.e., admission) weight from their self-reported highest past weight. Of 703 individuals eligible for TWS analyses, there were 100 cases of “negative WS,” wherein current weights were greater than reported highest past weights. In these cases, self-reported highest weight was replaced with current weight. While the average discrepancy was only 1.2 lb (*SD* = 6.5), suggesting small differences in rounding or normal weight fluctuations, the largest discrepancy was 94.2 lb.

After calculating TWS, a tertile split was used to derive low and high groups. This method was deemed appropriate based on prior findings by Lowe et al. (2011) in their sample with BN, which suggested that TWS levels in their bottom tertile of patients were indeed low, comparable to those found in individuals without EDs. Hereafter, the “TWS sample” will refer to those individuals captured within either the low or high tertile group according to TWS. Of note, because different variables were required for the respective calculations for TWS and DWS, sample sizes differed slightly. Furthermore, the resulting samples did not include all of the same participants. This non-overlap was largely due to individuals being categorized into either the low or high tertile according to one formulation, but the middle tertile according to the other.

**SM.2 Analytic Plan Using TWS**

Mixed-model ANOVAs were used to assess differences in weight history between low and high WS groups. All four TWS weight history variables (i.e., premorbid high weight, postmorbid low weight, postmorbid high weight, and admission weight) were winsorized to the 95^th^ percentile to address extreme outliers. Acceptable normality, skewness, and kurtosis were then confirmed for each variable. The assumption of sphericity was tested through Mauchly’s W and found to be violated (*p* < .001); therefore, our final results are reported with the Greenhouse-Geisser correction. Finally, Bonferroni post-hoc tests were conducted to probe for the source(s) of significant interactions.

**SM.3 TWS Sample Characteristics**

Table S1 shows participant characteristics for the TWS sample. Most participants (78.6%) were White. On average, participants presented to the clinic as adults (mean age of 25.3 years) and reported experiencing their first ED sign as adolescents (mean age of 14.6 years). Comparing low and high tertile groups on demographic variables resulted in significant differences among the TWS sample. Specifically, the low TWS group was found to present to treatment at an earlier age (23.4 years vs. 27.2 years), *t*(447.6) = -4.24, *p* < .001, and have a shorter length of stay (27.3 days vs. 29.8 days), *t*(464) = -2.25, *p* = .025, than the high TWS group. No significant differences were found on age of symptom onset between the low and high TWS groups (*p* = .20).

**Table S1**

*Participant characteristics for the TWS sample*

|  | TWS | | | |
| --- | --- | --- | --- | --- |
|  | *n* | Min | Max | *M* (*SD*) |
| *Age at admission* | 467 | 14 | 61 | 25.3 (9.9) |
| *Treatment length of stay, in days* | 466 | 2 | 99 | 28.6 (11.6) |
| *Age of symptom onset* | 467 | 4 | 38 | 14.6 (4.3) |
|  | | | | |
| *Ethnicity* | *n* (%) | | | |
| White | 367 (78.6) | | | |
| African American | 11 (2.4) | | | |
| Asian or Pacific Islander | 10 (2.1) | | | |
| Hispanic | 30 (6.4) | | | |
| Native American | 6 (1.3) | | | |
| Other | 15 (3.2) | | | |
| Multiracial | 21 (4.5) | | | |
| Total | 460 (98.5) | | | |

*Note*. Age of symptom onset was self-reported age at which first eating disorder sign was experienced, derived from the Dieting and Weight History Questionnaire (DWHQ). TWS = traditional weight suppression.

**SM.4 Weight History Analyses Using TWS**

A 2 x 4 mixed-model ANOVA was used to examine differences in weight history between low and high TWS groups. Raw weights were analyzed (rather than BMIs) to maintain consistency with the TWS formulation. However, *t*-tests confirmed that the two groups did not differ significantly on height at any of the four timepoints (all *p*s > .05), suggesting that results would not have differed substantially if BMI (i.e., weight adjusted by height) had been used in place of raw weights. Age was included as a covariate in a second ANOVA model, since low and high TWS groups differed significantly on age of admission; however, results were identical between the two models, so findings are reported without the covariate for simplicity.

The mixed-model ANOVA revealed a significant interaction between weight category (i.e., premorbid high, postmorbid high, postmorbid low, and admission) and tertile group (i.e., low TWS and high TWS), *F*(1.84,712.98) = 52.17, *p* < .001, *η_p_^2^* = 0.12 (see Figure S1). There were also significant main effects of both weight history category, *F*(1.84,712.98) = 328.25, *p* < .001, *η_p_^2^* = 0.46, and tertile group, *F*(1,387) = 4.69, *p* = .03, *η_p_^2^* = 0.01.

**Figure S1**

*Weight history by age for low and high TWS groups*


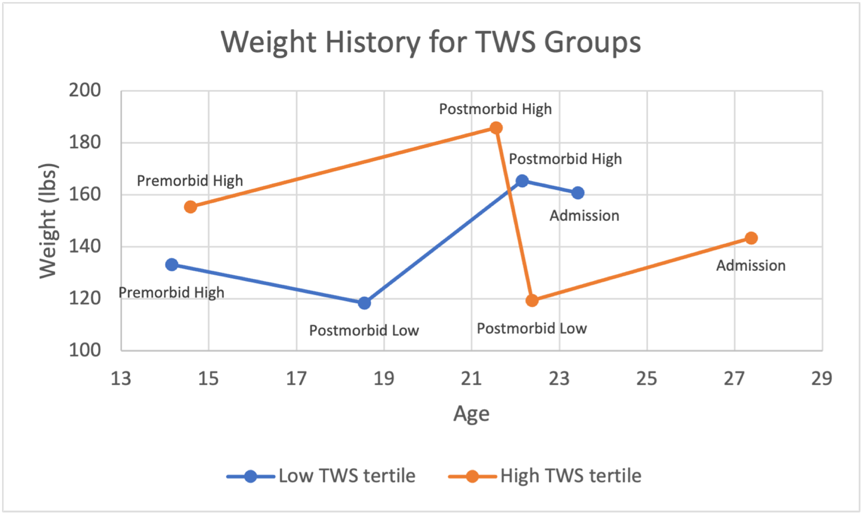


*Note*. Of those included in weight history analyses among the TWS sample (*n* = 389), age of symptom onset was 14.5 (*SD* = 3.8) for low TWS and 14.9 (*SD* = 5.0) for high TWS.

Within-group comparisons revealed that postmorbid low weights were significantly lower than premorbid high, postmorbid high, and admission weights for both low and high TWS groups (*p* < .001), and both groups surpassed their premorbid high weights postmorbidly (i.e., postmorbid high weights were significantly greater than premorbid high weights; *p* < .001). Additionally, for both groups, admission weights were significantly lower than postmorbid high weights (*p*s < .001). That is, both groups were weight-suppressed compared to their postmorbid high weights; however, the deficit was much greater for the high TWS group (mean difference of -42.5 lb) compared to the low TWS group (mean difference of -4.6 lb). Furthermore, only the high TWS group was significantly weight-suppressed compared to their premorbid high weight (mean difference of -12.0 lb; *p* < .001). In fact, the low TWS group presented to treatment at weights that were significantly *greater* than their premorbid high weights (mean difference of 27.8 lb; *p* = .037). Thus, while the high TWS group was significantly weight-reduced compared to both their premorbid high and postmorbid high weights upon admission, the low TWS group was only minimally weight-reduced compared to their postmorbid high weight and demonstrated weight *gain* compared to their premorbid high weight.

Between-group comparisons revealed that the high TWS group had significantly higher premorbid high and postmorbid high weights, but lower admission weights, than the low TWS group (*p’*s < .001; see Table S2), indicating that the relatively greater TWS of the high TWS group (compared to the low TWS group), came from *both* higher past weights and lower admission weights. Postmorbid low weights were not found to be significantly different between the two groups (*p* = .69).

**Table S2**

*Weight history comparisons (in lbs) between low and high TWS groups*

|  | Low TWS,  *M* (*SD*) | High TWS,  *M* (*SD*) | Mean Difference (Low - High) | *p* |
| --- | --- | --- | --- | --- |
| Premorbid high weight | 133.2 (29.2) | 155.4 (39.6) | -22.2 (3.5) | <.001 |
| Postmorbid high weight | 165.5 (44.5) | 185.9 (51.4) | -20.4 (4.9) | <.001 |
| Postmorbid low weight | 118.4 (23.8) | 119.4 (23.2) | -0.9 (2.4) | .69 |
| Admission weight | 160.9 (40.6) | 143.4 (33.5) | 17.5 (3.8) | <.001 |

In post-hoc analyses comparing TWS groups on age at which each weight occurred, we found a significant interaction between age category (i.e., premorbid high, postmorbid high, postmorbid low, and admission) and tertile group, *F*(2.18,826.39) = 19.70, *p* < .001, *η_p_^2^* = 0.05*,* as well as significant main effects of age category, *F*(2.18,826.39) = 305.63, *p* < .001, *η_p_^2^* = 0.45, and tertile group, *F*(1,380) = 9.20, *p* = .003, *η_p_^2^* = 0.02.

Bonferroni post hoc analyses revealed, unsurprisingly, that premorbid high weights were experienced at significantly younger ages, and admission weights at significantly older ages, than all other weight categories for both groups (*p*s < .001). However, within the low TWS group, postmorbid low weights were experienced significantly earlier (*M* = 18.5 years, *SD* = 6.3) than postmorbid high weights (*M* = 22.2 years, *SD* = 8.4; *p* < .001); in contrast, within the high TWS group, postmorbid low age (*M* = 22.4, *SD* = 8.0) and postmorbid high age (*M* = 21.6, *SD* = 7.5) were not significantly different from each other (*p* = .60). Again, whereas a vast majority of participants within the low TWS group (74.7%) reported experiencing their postmorbid low weights first, relative to their postmorbid high weights, a majority of participants within the high TWS group (64.4%) reported experiencing their postmorbid high weights first.

**SM.5 Differences Between Analyses Using TWS and Analyses Using DWS**

The TWS and DWS formulations resulted in partially distinct samples. That is, of the 467 participants in the TWS sample, 147 were not represented in the DWS sample, and of the 453 participants in the DWS sample, 133 were not represented in the TWS sample. Although this non-overlap was partially due to missing data (i.e., of the original 709 participants, 31 were eligible for TWS analyses but not DWS analyses, and 6 were eligible for DWS but not TWS analyses), it was largely due to different individuals being captured within the low and high tertile groups according to the two formulations. In other words, a substantial portion of individuals categorized as being in either the bottom or top third percentile of WS according to one formulation fell into the middle tertile according to the other formulation (and, thus, were not included in the respective analyses). Furthermore, some individuals categorized as having *high* TWS actually fell into the *low* DWS tertile, and vice versa. Therefore, differences in findings between the two formulations may also partially result from the fact that the samples did not include the same individuals within their low and high tertile groups.

While the pattern of results found when using TWS was similar to the pattern found when using DWS, there were some minor differences. First, while both high and low TWS groups surpassed their premorbid high BMIs postmorbidly, only the low DWS group surpassed their premorbid high z-BMI postmorbidly. The high DWS group, on the other hand, had a postmorbid high z-BMI that was significantly lower than their premorbid high z-BMI. Second, while postmorbid low weights did not differ significantly between low and high TWS groups, the high DWS group had a postmorbid low z-BMI that was significantly lower than the low DWS group. Third, while the high TWS group had a *higher* postmorbid high weight than the low TWS group, the high DWS group had a *lower* postmorbid high weight than the low DWS group. Finally, differences emerged between TWS and DWS groups on age at which low and high tertiles presented to treatment. That is, whereas the low TWS group presented at a significantly earlier age than the high TWS group, there was no difference between the DWS groups.
